# Increasing the Reliability of Data Collection of Laser Line Triangulation Sensor by Proper Placement of the Sensor

**DOI:** 10.3390/s21082890

**Published:** 2021-04-20

**Authors:** Dominik Heczko, Petr Oščádal, Tomáš Kot, Daniel Huczala, Ján Semjon, Zdenko Bobovský

**Affiliations:** 1 Department of Robotics, Faculty of Mechanical Engineering, VSB-Technical University of Ostrava, 70800 Ostrava, Czech Republic; petr.oscadal@vsb.cz (P.O.); tomas.kot@vsb.cz (T.K.); daniel.huczala@vsb.cz (D.H.); zdenko.bobovsky@vsb.cz (Z.B.); 2 Department of Robotics, Faculty of Mechanical Engineering, Technical University of Kosice, 04200 Kosice, Slovakia; jan.semjon@tuke.sk

**Keywords:** angle of incidence, AoI, incidence angle, laser scanner, laser intensity, surface properties, transparent, shiny, glossy surface, sensor placement

## Abstract

In this paper, we investigated the effect of the incidence angle of a laser ray on the reflected laser intensity. A dataset on this dependence is presented for materials usually used in the industry, such as transparent and non-transparent plastics and aluminum alloys with different surface roughness. The measurements have been performed with a laser line triangulation sensor and a UR10e robot. The presented results are proposing where to place the sensor relative to the scanned object, thus increasing the reliability of the sensor data collection.

## 1. Introduction

Laser scanning is a widely used process in both industrial and non-industrial environments. In non-industrial sectors, these are terrestrial laser scanning and airborne laser scanning methods that use received intensity data as the main data source in various studies, such as vegetation and forest exploration [1], scanning and creating 3D models of buildings [2]. In an industrial environment, laser scanning is mainly used for reverse engineering [3,4], component localization systems for bin-picking applications [5], product inspection, checking dimensions and quality [6,7,8], or control of the production process, e.g., inspection of welds in robotic welding [9].

In the industrial environment, laser line triangulation (LLT) sensors, which operate on the principle of triangulation [10,11], are commonly used. The already mentioned inspection of the components can also be carried out by contact sensors, but the LLT sensors have advantages in speed and resolution of the measurement. Contactless measurements cannot damage the checked component in any way. In addition, 3D point clouds can be generated using LLT sensors for further processing. On the other hand, LLT sensors can be affected by the geometry and reflective properties of scanned surfaces that can cause distortion.

Another limiting factor is shiny and smooth surfaces which cause specular reflections. Specular reflection depends on material reflectance, surface roughness, and geometric scanning parameters such as the incidence angle of a laser ray (beam), and the out-plane and in-plane angle of a sensor [12,13].

Transparent surfaces are problematic to scan because of the refraction problem [14]. For transparent surfaces, the laser beam is split and reflected from both the upper and the lower surface. The sensor can detect both beams; however, this is causing disturbances. Nonetheless, there are methods allowing scanning transparent surfaces using area filtering masks or by detecting UV light [15].

**Figure 1:**
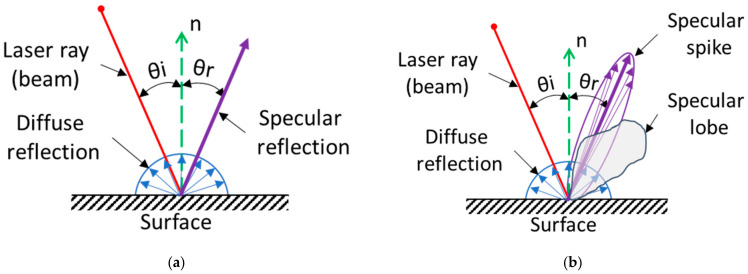
Reflection phenomenon. For an ideal specular reflection, the angle of incidence (AoI) θ_i_ is the same as the angle of reflection θ_r_ relative to the surface normal n; (**a**) the general distribution of the reflection; (**b**) the distribution of the reflection by [23].

The digitization of specular glossy surfaces is still problematic even though it has undergone many advances; e.g., in [16], the authors presented a method for the reconstruction of a specular surface, using a single camera viewpoint and the reflection of a planar target placed at two different positions; in [17], the authors projected several patterns on the shiny object, and the reflections of these patterns were processed and used for geometry reconstruction of the scanned object; other methods can be found in [18], which is a review on this topic. The diffusion intensity level, shown in Figure 1 of the shiny surfaces captured by the sensor was very small, leading to poor data collection [19]. In addition, specular reflections led to a greater occurrence of outliers, which were points located outside the captured cloud of points, which could be considered as noise. However, these outliers could be eliminated by various methods, e.g., in [12], the authors examined the effect of scanning orientation on the formation of outliers to facilitate their detection and subsequent removal; in [20], the authors presented a toolkit for cleaning point clouds.

Reflectance is the ability of a material to reflect light, which depends on the optical properties of that material. A beam of light can be reflected in two ways (types) as shown in Figure 1a. Diffuse and specular reflection [21]. Both types of reflection exist at the same time, though one of them dominates. For glossy materials, specular reflection prevails, and for matte materials, diffusion reflection prevails. It is the diffusion reflection that is desirable for laser scanning; the ideal surface with a high light-scattering index is referred to as the Lambertian surface [22].

According to [23], we can divide the reflection of the beam of light into three types of reflection, as seen in Figure 1b. Diffuse reflection, specular spike, and specular lobe. The type of reflection depends on the material, its reflective properties, and the roughness of the surface: With increasing roughness, the specular spike is shrinking and the specular lobe is increasing [23].

Some studies investigated the relationship between surface roughness and laser scanning. In [24,25], authors analyzed the influence of the surface roughness on the scanning quality and accuracy. They proved that the quantity of detected points and accuracy is influenced not only by the roughness itself but also by the manufacturing process, such as horizontal and vertical milling or turning [26].

Additionally, ambient light causes noise in captured data (point clouds), which has an impact on the detection accuracy of this data [27]. According to [19], when ambient light is inhibited during scanning, the collected data contain a higher number of points while the noise level is lowered. If the manufacturing process requires lighting, one can choose a source of light that provides a more reliable acquisition of scanned data, as studied in [28].

Another option for scanning shiny and transparent surfaces is an application of an additional layer of matte material to the scanned object. Using this method, the intensity of the specular reflection is significantly reduced and the diffuse scattering of light into the surroundings is strengthened, which is desirable for detection by the sensor. However, this method is not always possible, as the applied material can devalue the surface of the part (e.g., the remains of a sticky part or spray, etc.). In addition, this added material must be removed, which requires an additional process that extends the production and control (inspection) times and increases production costs.

In this work, we investigated the effect of the incidence angle of a laser ray (beam) on this laser intensity. We presented results for this relation for industrial construction materials such as non-transparent colored plastics, transparent colored and pure plastics, and aluminum alloy with different surface roughness. These samples have a glossy, transparent, or matte surface. Our objective was to introduce the possibility of scanning these surfaces by an LLT sensor without adding matte materials to the surface of the scanned object. The acquired functions of the incidence angle allowed us to place the sensor in a position and orientation that increases the reliability of the data acquisition. Furthermore, these characteristics can also be implemented in simulation programs for virtual laser scanning and thus simulate different material behavior.

## 2. Methodology

**Figure 2:**
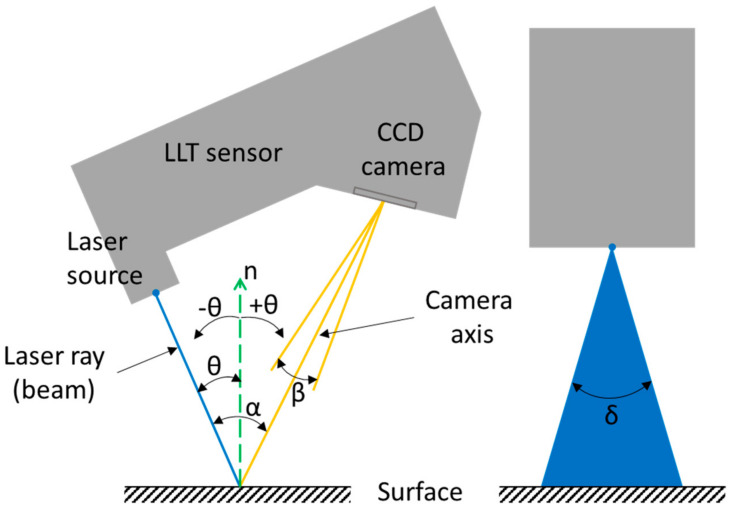
LLT sensor schematic. The α angle is the angle between the laser beam and the camera axis (triangulation angle); β is the vertical field of view of the CCD camera; δ is the angle of the cone of the projected laser. α, β, and δ are constants, which is given by the sensor manufacturer. θ is the AoI of the laser rays (beams) plane relative to the surface normal (n).

For sensors that have a laser transmitter and receiver in the same location, the AoI of the laser ray (beam) is always positive. In our case, we also considered a negative value of the AoI, because the CCD camera (receiver) is not located in the same location as the transmitter (laser source). The positive and negative directions of the AoI and sensor schematic are shown in Figure 2; the CCD camera is detecting the laser line at a fixed angle.

**Figure 3:**
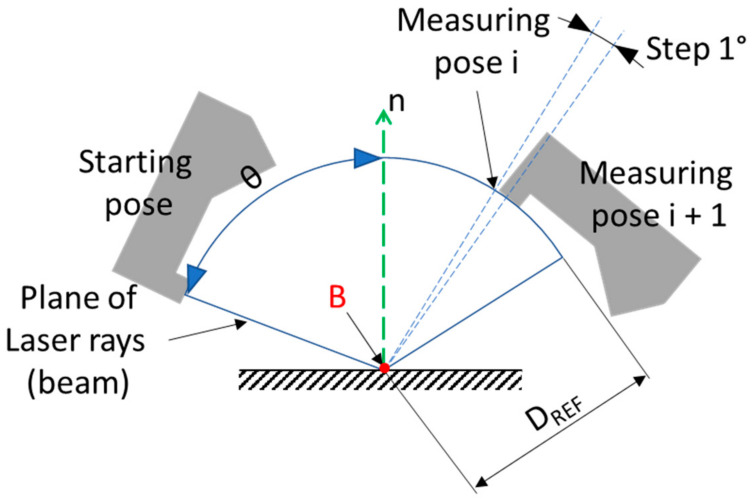
Schematic of the experimental measurement. D_REF_ is the reference distance of the sensor (Z-axis), θ is the AoI of the laser rays (beams) plane relative to the surface normal (n). Point B represents the laser line (it is perpendicular to the image) around which the robot rotates the LLT sensor.

Section 3 describes the measuring process and data analysis in details. To collect the data for evaluation of the influence of AoI on the reflected laser intensity, we performed the steps visualized in Figure 3. The robot moved the LLT sensor to the starting pose and then subsequently rotated around the laser line (visualized as the red point B in Figure 3) by 1°, which represented a measuring pose. In total, 140 poses from the AoI θ −85° to +55° were measured.

The objective of this study was to determine the interval of positioning and orientation of the sensor with respect to the surface. The parameters to decide which interval provided the most reliable data were the detected number of points and the intensity level.

## 3. Experiment Setup and Details

Rotary collaborative robot UR10e (basic parameters shown in Table 1) was used to handle the LLT sensor. Using a robot with six degrees of freedom (DoF), the sensor can be positioned exactly in the desired position and orientation towards the measured object without the production of complex jigs or positioning mechanisms.

The LLT sensor is mechanically attached to the robot using an aluminum L profile. The LJ-X8080 (provided by Keyence) sensor with parameters shown in Table 2 was used for this measurement.

**Figure 4:**
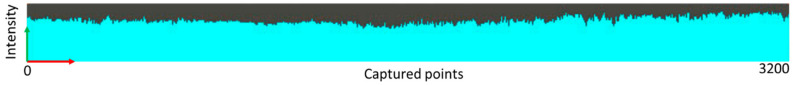
An example of raw intensity data for the full profile (3200 points) provided by the sensor control unit. The X-axis represents captured points, Y-axis represents intensity from 0 to 10 mW.

The sensor was controlled by the LJ-X8000e control unit (by Keyence), which allowed us to measure the laser intensity throughout the whole profile of points (for each point). Figure 4 shows the example of raw intensity data provided by the sensor control unit for one full profile (3200 points); the X-axis represents the captured points; the Y-axis represents the light intensity of the laser for each point, measured by a charge-coupled device (CCD) chip camera inside of the LLT sensor.

### 3.1. Experimental Workplace

**Figure 5:**
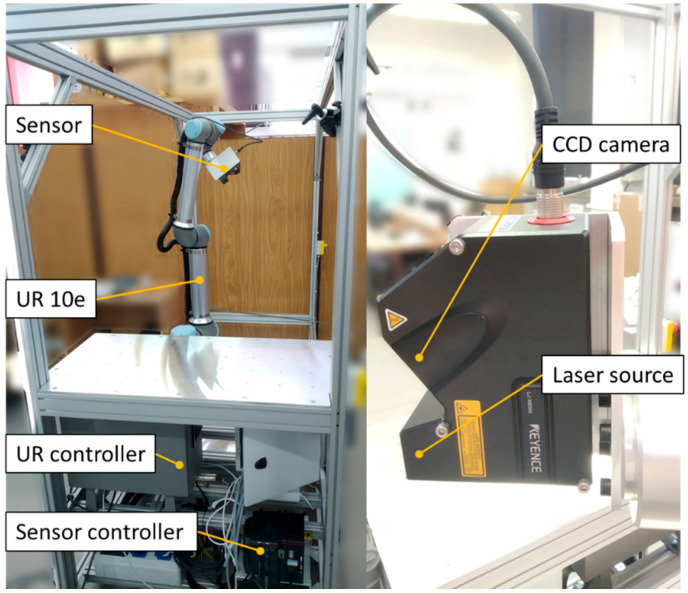
Experimental workplace.

The workplace was made of aluminum profiles on which an aluminum plate with a matrix of threaded holes was mounted. The UR10e robot was attached to this plate. Due to the threaded holes in the matrix, we could easily attach various holders in the exact positions relative to the robot. The configuration of the workplace can be seen in Figure 5.

**Figure 6:**
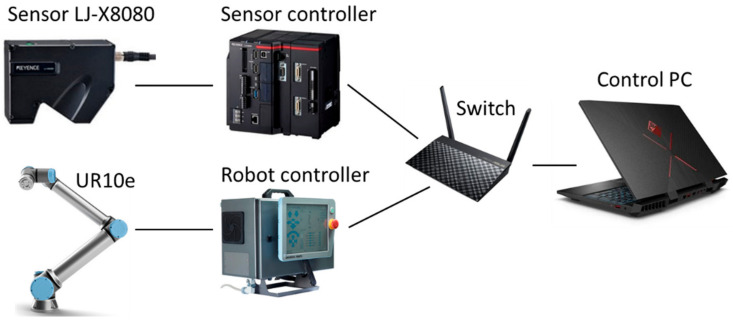
Communication diagram of the workplace.

The workplace was controlled by a C# application. For fast communication, the controllers of the robot and sensor were connected to an Ethernet switch and then to the control computer via an Ethernet connection. Communication was done via TCP/IP protocol. The communication diagram can be seen in Figure 6.

**Figure 7:**
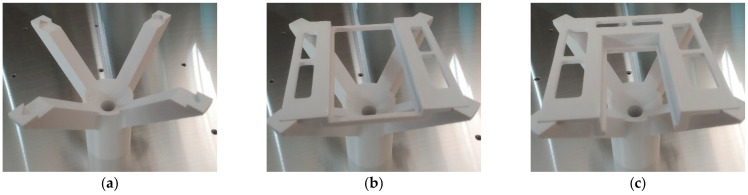
Plastic holder for scanning samples of thickness: (**a**) 3; (**b**) 5; (**c**) 12 mm.

The scanned samples had different dimensions and thicknesses (as seen in Table 3). The robot always performed the same moves for the scanning of all samples, as previously shown in Figure 3. There was a plastic holder with attachments that held the scanned surface at the same level, as can be seen in Figure 7a–c. The holder was attached to the threaded hole of the matrix, so its position against the robot was known.

**Figure 8:**
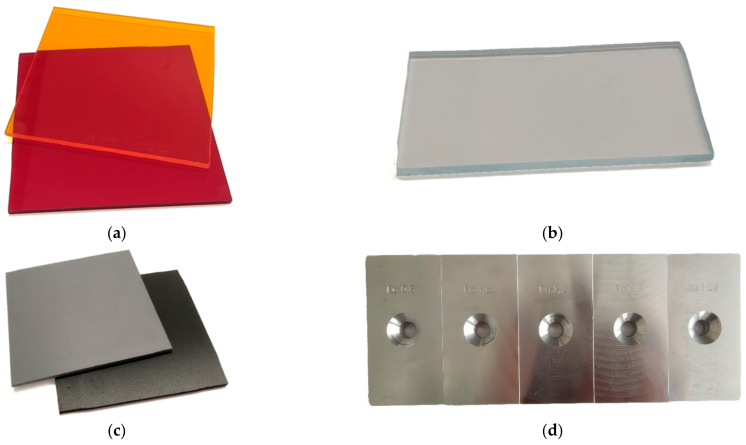
Scanned samples: (**a**) Transparent colored plastics (acrylic sheets); (**b**) transparent pure plastic (acrylic sheet); (**c**) non-transparent colored plastics (PVC plastics); (**d**) aluminum alloy with different roughness (Ra0.8–12.5).

The materials of the samples for measuring the function of the AoI were the commonly used materials in automotive. These were red and orange transparent plastics (Figure 8a), transparent clear plastic (Figure 8b), a black plastic plate, and a gray plastic plate (Figure 8c), and an aluminum alloy with different surface roughness (Figure 8d), made by milling. To easily determine the AoI of individual laser rays, all samples were flat.

### 3.2. Data Collection

The robot moved the sensor to the desired position and orientation above the sample (scanned material) so that the measured displacement of the sensor was equal to its reference distance. The LLT sensor projected a laser line to the scanned object, which served as the axis of rotation when rotating the sensor head of the robot. In this way, the robot gradually rotated the sensor from the AoI θ −85° to +55° with a step of 1°. This angle range was the maximum possible scanning range of the LJ-X8080 sensor. By rotating the sensor outside the specified interval, the projected laser line would be no longer detected by the CCD camera. In each rotation position, data were collected and stored for further processing. The measurement process was described by Algorithm 1.
**Algorithm 1.** The measurement process.**for** i = 0 to m   // m[1:140] represents the position (pose) number, each position corresponds to a specific AoI        **set_robot_pose** (i)       **waittime**  // stabilization of the robot       **for** j = 0 to n   // n[1:30] represents the number of frames in one position               Get profile data               Get image data               Save data     **end for**     Get mean profile data     Get mean intensity data **end for**

The robot was not 100% rigid short time after stopping in a position. There was so-called shifting, which was a slight displacement of the tool center point of the robot. Therefore, in each position, the data were measured 30 times with an interval of 250 ms to minimize the influence of shifting and vibrations caused by the movement of the robot. The intensity data obtained for each point were averaged and then normalized to a range of 0 to 1. The mean intensity value for one point was calculated by Equation (1):

(1) $$\bar{I_{n}}=\frac{1}{n}\overset{n}{\underset{i=1}{\sum }}I_{i}$$

where $\bar{I_{n}}$ is the mean intensity value of one point in a measured pose and $I_{i}$ is the normalized intensity value of one point in a measured pose of the i-th scan. The normalized mean intensity value was used for further processing.

**Figure 9:**
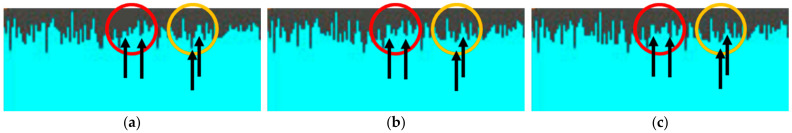
Raw intensity data in a part of the scanned profile. Differences of measured intensity at one pose: (**a**) Scan no. 4; (**b**) scan no. 14; (**c**) scan no. 16.

When data from multiple measurements were compared, no significant variability was immediately obvious. However, minor differences of measured intensity at one pose of the sensor can be observed in Figure 9 with a detailed look. In Figure 9a, the measured intensity was influenced by shifting and robot vibrations, while in Figure 9b,c the intensity value was more stable, but we could still observe small differences that are marked by circles (the area marked by the red circle in Figure 9a corresponds to the areas marked by the red circle in Figure 9b,c; the same convention goes for orange circles).

**Figure 10:**
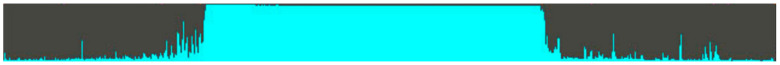
The measured intensity of one profile on a shiny smooth surface. Points in the laser line are no longer detected on the left and right side because of low laser intensity.

The saved profile may not have contained all points, and a smaller or larger portion may have been missing. Such a situation occured when the sensor did not detect enough reflected light from the target, mostly from shiny smooth surfaces on which specular reflection dominated. An example of an incomplete profile can be seen in Figure 10, where the side laser rays are no longer detected by the CCD camera.

**Figure 11:**
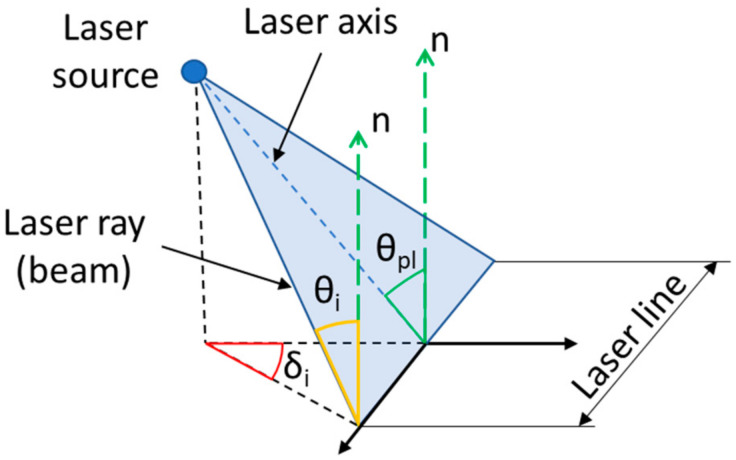
Schematic of the laser ray geometry for computation of AoI of i-th laser ray.

The data processing procedure describes Algorithm 2, which worked with averaged intensity data. AoI of each laser ray was calculated by Equation (2):

(2) $$\theta_{i}=\sqrt{\theta_{\mathrm{pl}}^{2}+\delta_{i}^{2}}$$

where $\theta_{\mathrm{pl}}$ is the AoI of the laser ray plane, and $\delta_{i}$ is the AoI of the corresponding beam in the projected cone, as shown in Figure 11. Subsequently, the appropriate intensity was assigned to each detected point.
**Algorithm 2.** Acquisition of reflected intensity data.**for** i = 0 to m  // m[1:140] represents a saved scan that corresponds to a certain pose of the sensor        **for** j = 0 to n  // n[1:3200] represents the point ID number in the saved profile              **if** n exists                      **get_angle_of_incidence** (j)                      **assign_intensity_to_point** (j)              **end if**        **end for**        Save data **end for**


## 4. Results

**Figure 12:**
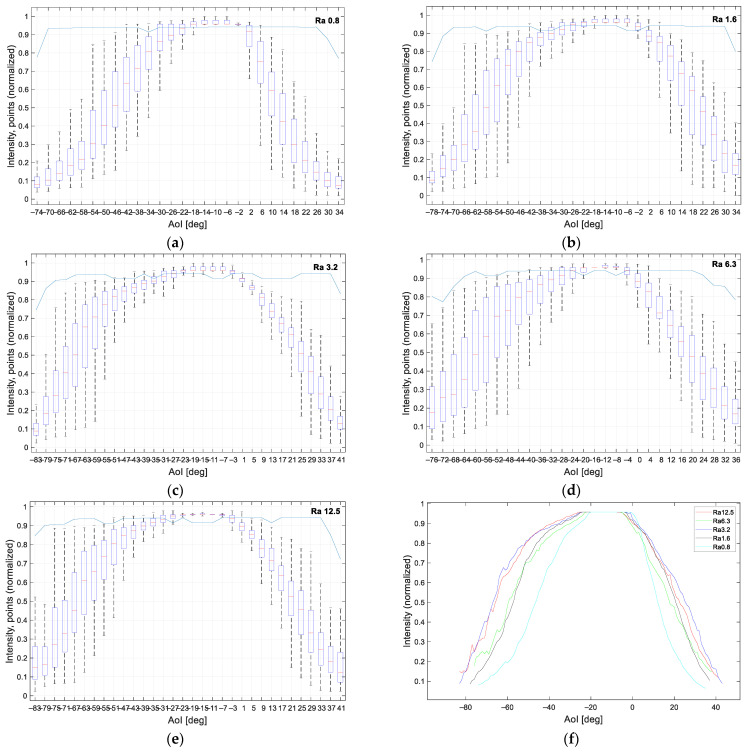
Processed intensity of aluminum alloy of different roughness: (**a**) Ra0.8; (**b**) Ra1.6; (**c**) Ra3.2; (**d**) Ra6.3; (**e**) Ra12.5; (**f**) median values in each measured position for all aluminum samples.

The incidence angle dependencies are shown in the following graphs in which the intensity was normalized as a value from 0 to 1. The intensity of detected points in one profile for each AoI was plotted using boxplots. The same plots also contained a blue line representing the percentage of the detected points. For better clarity, the graphs were cropped on sides and did not show data when no laser reflection was detected. For example, the sample in Figure 12a was detected from −74° to 34°. The step of 4° was set for aluminum samples; for plastic non-transparent samples the detected interval was greater, therefore the data were plotted with a step of 5°.

### 4.1. Aluminum Alloy with Different Roughness

The intensity for aluminum samples with different roughness is plotted in Figure 12a–e. For rough surfaces (e.g., Ra12.5), the laser line was detected at AoI −83° to +41°, while for smooth surfaces (e.g., Ra0.8) the laser line was detected only at AoI −74° to +34°. The measured intensity data were very similar to each other. In Figure 12f, there is the median intensity of all measured positions. We could observe the sharp intensity change in relation to the angle of incidence for the sample with surface roughness Ra0.8. For smaller roughnesses (Ra0.8–1.6), the graph of the median intensity was smooth; for higher roughness, we could observe step changes in intensity, which was due to the irregularities on the material surface and manufacturing process [25,26].

### 4.2. Transparent Plastics

For the clear transparent material, both the bottom and top surface of the material was detected, which was undesirable in most applications, and therefore an area filtering mask had to be applied to eliminate detection of unwanted surfaces.

**Figure 13:**
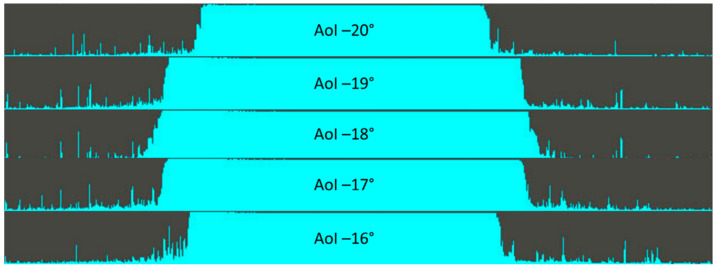
Raw intensity data for orange plexiglass. The other transparent plastics have similar intensity.

For all transparent materials, the laser line was detected only at AoI −20° to −16° and even at these incidence angles, the profile of points was not detected completely. Only about 50% of points was detected in a profile, as shown in Figure 13, which represented raw intensity data AoI −20° to −16°. This area of incidence angles represented specular reflections; therefore, the measured intensity (normalized) was either one or almost zero.

**Figure 14:**
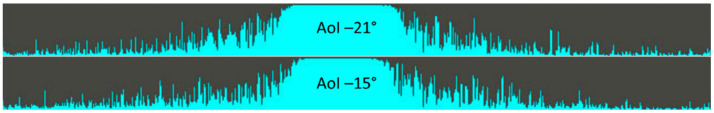
Raw intensity data for red plexiglass at AoI −21° and −15°. Points in the laser line are no longer detected on the left and right side because of the low laser intensity; we can also observe the noise which leads to a high occurrence of outliers.

In the red plexiglass, the laser line was also detected at AoI −21° and −15°, as shown in Figure 14. However, only about 20% of points in the profile were detected in these positions, and significant noise could be seen outside the detected points.

For transparent materials, the processed data are shown in Table 4, Table 5 and Table 6 instead of boxplots, due to specular reflections. The median, the twenty-fifth percentile, and the seventy-fifth percentile had the same value in the area of specular reflection, which was the AoI of the laser beam from −20° to −16°; except for the red material, where the twenty-fifth percentile was affected by noise (outliers).

**Figure 15:**
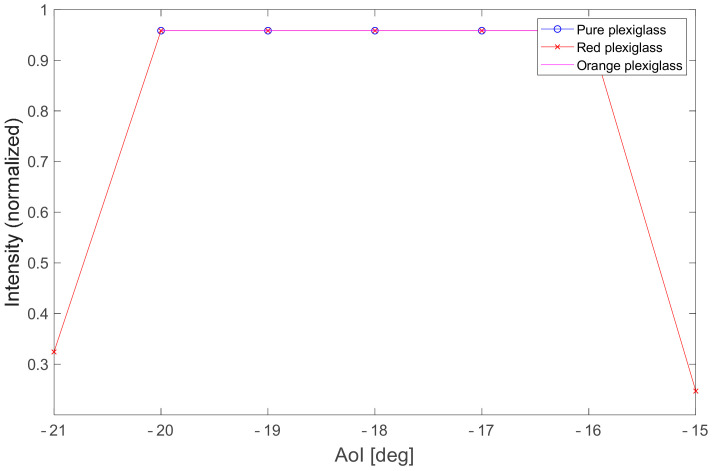
Median values of intensity in measured positions of transparent plastic samples.

The graphs of the median intensity for transparent plastics can be seen in Figure 15; the laser line was detected only in the area of specular reflection.

### 4.3. Non-Transparent Plastics and Overview

**Figure 16:**
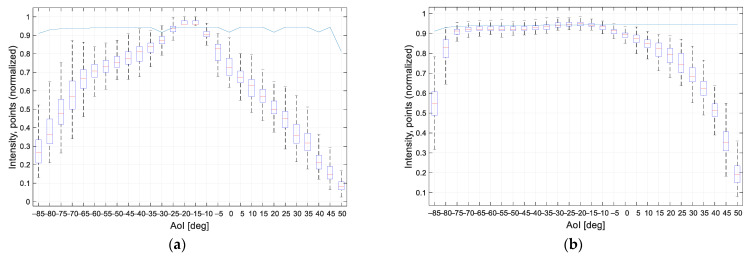

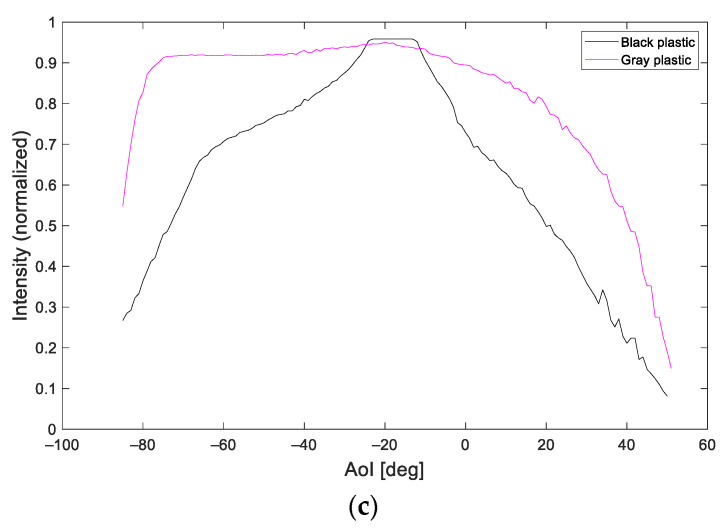
The processed intensity of non-transparent plastics of different colors: (**a**) Black; (**b**) gray; (**c**) median values in each measured position for non-transparent plastic samples.

The intensity for the black plastic was plotted in Figure 16a and for the gray plastic in Figure 16b. For both materials, the laser line was detected at AoI −85° to +50°. Although it is the same material with the same surface roughness, we can observe a different shape of the intensity graph. In this case, the color of the material played an important role, where the black object absorbed part of the light beam [29]. The graphs of the median intensity are shown in Figure 16c.

### 4.4. The Results Overview

**Figure 17:**
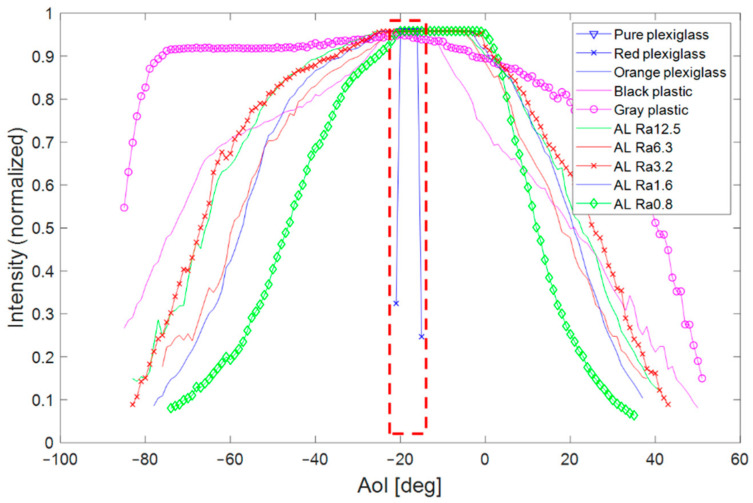
Dependence of the laser intensity on the angle of incidence for all samples; the specular reflection area was marked by a red rectangle with a dashed line.

The functions of the incidence angle on laser intensity for all materials are shown in Figure 17, where the red rectangle with the dashed line shows the specular reflection area. Only in this area was the laser line detected on transparent and shiny surfaces. In this area, the greatest laser intensity was detected in other materials as well. A diffusion reflection was in the area outside the red rectangle.

In Table 7, there is a list of the detection range of the laser line for each material. In addition, for each material, there was defined an interval of AoI with high intensity corresponding with a high number of detected points. In the case of non-transparent materials, we chose intervals where both values were above 90%. For transparent plastics, the recommended range was the same as the whole detection range where only 50–70% of the points were being detected, as explained in Section 4.2. This interval was recommended for setting the position and orientation of the sensor relative to the scanned object.

## 5. Conclusions

Laser scanning (1D, 2D, or 3D) is a commonly used technology in the industry; whether for surface quality control, checking dimensions and quality, or creating point clouds for object localization and further processing. To achieve accurate results, it is essential to obtain reliable data from the sensor.

In this article, we focused on examining the effect of the laser beam incidence angle on the laser intensity. Data were measured by an LLT sensor, which is commonly used in the industry. This angle function on laser intensity was investigated on various materials that are frequently used in automotive (red, orange, and clear transparent plastics, black and gray plastic, and aluminum alloys with different roughness). Of course, more materials can be scanned (e.g., different steel alloys, leathers), and the proposed methodology for obtaining these functions can be used for these materials as well.

The appropriate intensity for laser scanning was the highest intensity of diffusion reflection. For aluminum samples, the maximum intensity was measured at the AoI interval [−25°, 0°]; but the laser line was already detected at AoI from −83° to +41° for samples with higher surface roughness; for samples with lower roughness (smoother surface), the laser line was detected at AoI from −74° to +34°.

For the black plastic sample, the maximum intensity was noticed at the AoI interval [−24°, −12°]. Outside of this interval, the intensity of the laser changed significantly with the changing AoI. In this case, the color of the material played a big role, where the black color absorbed light. For a gray sample, the highest intensity was observed at the AoI interval [−80°, 0°]; for AoI 0° and above, the laser intensity decreases continuously. Therefore, the gray sample was well scanned at a wide range of angles. For both samples, the laser line was detected at AoI from −85° to +50°.

For transparent plastics, the laser line was detected only at the AoI interval [−20°, −16°], which was the specular reflection area. Obtained profiles with these AoI did not contain all points, but only 50%, as seen in Figure 13. These surfaces were very smooth (almost mirror-like), where specular reflection dominated, and the diffuse light scattering was almost zero. The outer beams of the scanning profile (on the left and right side) were reflected outside the CCD camera and were therefore not detected. In addition, while scanning pure transparent plastic, there was a refraction problem where both the top and bottom surfaces were detected. In our case, the reflection from the lower surface was undesirable and was eliminated using an area filtering mask. However, for some applications, detection of both surfaces was required and was used, for example, to measure the thickness of the material.

Presented data showed that each material had different reflective properties. As seen in Figure 17, the laser line was detected under different AoI for each material. Therefore, the measured dependence of the laser intensity on the AoI could help to correctly place the LLT sensor in the production or control process for more reliable data collection. These characteristics could also be used as data in the virtual scanning simulation environment to improve the sensor location (position and orientation) relative to the scanned object, or to test scanning by the LLT sensor before a real sensor was bought.

The proposed method brought benefits for practical usage in industry. The process of design and deployment of a workplace could be sped up and its cost lowered when the placement of LLT sensor providing reliable data was known, so there is no need for additional tests and experiments with different hardware options. The workplaces already participating in a manufacturing process could be adjusted to achieve even better results. Furthermore, this methodology could be transferred even to mobile robotics. For example, in simultaneous localization and mapping (SLAM), laser scanning is often used to localize a robot or to inspect the environment. The correct positioning of such robot in respect to scanned objects may increase the accuracy and reliability of the detection.

For future research, this methodology can be extended to exterior materials used in automotive either before painting (sheet metal) or after painting (different colors of the painted layer), so the list of suggested measuring angles would contain other materials. Furthermore, this methodology could be extended by checking the quality of the obtained point cloud, that is, whether the points are distributed according to a plane by measuring the dispersion of the points on that plane.

## Figures and Tables

**Table 1:** Basic parameters of the UR10e robot.

Parameter	Specification
DoF	6
Reach	1300 mm
Payload	10 kg
Pose repeatability	±0.05 mm

**Table 2:** Parameters of LJ-X8080 sensor.

Parameter	Specification
Reference distance (Z-axis)	73 mm
Triangulation angle	35°
Measuring range (Z-axis)	±20.5 mm (full scale = 41 mm)
Measuring range (X-axis)	30 mm (near side)
	35 mm (reference distance)
	39 (far side)
Linearity (Z-axis)	±0.03% of the full scale
Profile data count	3 200 points
Laser type	Blue laser
Laser source	10 mW
Laser wavelength	405 nm (visible light)

**Table 3:** Dimensions, surface roughness, and flatness of scanned objects.

Material	Width [mm]	Height [mm]	Thickness [mm]	Roughness [µm]	Flatness [mm]
Non-transparent plastics	100	100	3	Ra1.2	0.02
Colored transparent plastics	100	100	3	Ra0.01–0.04	0.01
Pure transparent plastic	50	100	5	Ra0.01–0.04	0.01
Aluminum alloy	40	80	12	Ra0.8, 1.6, 3.2, 6.3, 12.5	0.02

**Table 4:** Processed intensity data for red transparent plastic.

AoI	Median	Mean	25th Percentile	75th Percentile
−21°	0.324	0.445	0.133	0.875
−20°	0.958	0.597	0.158	0.958
−19°	0.958	0.691	0.259	0.958
−18°	0.958	0.716	0.320	0.958
−17°	0.958	0.675	0.241	0.958
−16°	0.958	0.633	0.191	0.958
−15°	0.310	0.428	0.134	0.785

**Table 5:** Processed intensity data for orange transparent plastic.

AoI	Median	Mean	25th Percentile	75th Percentile
−20°	0.958	0.815	0.958	0.958
−19°	0.958	0.880	0.958	0.958
−18°	0.958	0.854	0.958	0.958
−17°	0.958	0.865	0.958	0.958
−16°	0.958	0.822	0.958	0.958

**Table 6:** Processed intensity data for pure transparent plastic.

AoI	Median	Mean	25th Percentile	75th Percentile
−20°	0.958	0.929	0.958	0.958
−19°	0.958	0.961	0.958	0.958
−18°	0.958	0.960	0.958	0.958
−17°	0.958	0.962	0.958	0.958
−16°	0.958	0.961	0.958	0.958

**Table 7:** Detection range of the laser line for all materials. The recommended range provides the highest reflection intensity, based on the experimental measurements.

Material	Detection Range	Recommended Range
Aluminum alloy Ra0.8	[−74°, 34°]	[−21°, 0°]
Aluminum alloy Ra1.6	[−78°, 34°]	[−25°, −1°]
Aluminum alloy Ra3.2	[−83°, 41°]	[−26°, 0°]
Aluminum alloy Ra6.3	[−76°, 36°]	[−24°, −3°]
Aluminum alloy Ra12.5	[−83°, 41°]	[−30°, −1°]
Pure transparent plastic	[−20°, −16°]	[−20°, −16°]
Orange transparent plastic	[−20°, −16°]	[−20°, −16°]
Red transparent plastic	[−21°, −15°]	[−20°, −16°]
Black non-transparent plastic	[−85°, 50°]	[−25°, −11°]
Gray non-transparent plastic	[−85°, 50°]	[−80°, 0°]

## Data Availability

Contact correspondence author.

## Abbreviations

The following abbreviations are used in this manuscript:

AoI	Angle of incidence
LLT	Laser line triangulation
CCD	Charge-coupled device
DoF	Degrees of freedom
